# Two Cases of Accidental Injection of Epinephrine into a Digit Treated with Subcutaneous Phentolamine Injections

**DOI:** 10.1155/2013/586207

**Published:** 2013-08-05

**Authors:** Ryan P. Bodkin, Nicole M. Acquisto, Holly Gunyan, Timothy J. Wiegand

**Affiliations:** ^1^Department of Emergency Medicine, University of Rochester Medical Center, 601 Elmwood Avenue, Box 655, Rochester, NY 14642, USA; ^2^Department of Pharmacy, University of Rochester Medical Center, 601 Elmwood Avenue, Box 638, Rochester, NY 14642, USA

## Abstract

Accidental injection into the digit from an epinephrine autoinjection device can cause discoloration, pain, and paresthesias. Although loss of digit is rare, treatment in the emergency department is commonly aimed at vasodilation of the affected tissue. We report two cases of accidental injection of epinephrine into the digits that were successfully treated with subcutaneous phentolamine injection with no adverse events.

## 1. Introduction

An intramuscular injection of epinephrine is currently the gold standard of treatment for severe anaphylaxis reactions. Autoinjection devices (i.e., EpiPen and Twinject) have been commercially available for the treatment of self-diagnosed anaphylaxis in the outpatient setting for over 30 years. Although education is provided to patients on the proper administration of these devices, the anxiety of the acuity of an anaphylaxis event compounded by the infrequency of use leads to accidental digital injections during administration. It is reported that this occurs in approximately one in 50,000 attempts at injection [1]. The Ruth Lawrence Poison and Drug Information Center covering the Finger Lakes Upstate Region of New York (14 counties) reports 27 cases of accidental injection over the past 10 years, and the Upstate New York Poison Center (54 counties) reports a total of 48 cases in 2011 making this a rare presentation. The prescribing information advises patients to go to the emergency department immediately for treatment directed at vasodilation if there is inadvertent administration to the digits, hands, or feet [2]. 

Epinephrine causes severe vasoconstriction through agonism of alpha_1_ and alpha_2_ receptors on the vascular smooth muscle. Based on this mechanism, it is believed that a direct injection of concentrated epinephrine 1 : 1,000 into the digit could lead to ischemic necrosis of the area. However, there is a lack of available reports showing that this in fact occurs [3–5]. Since symptoms including digital pain and paresthesias are reported to last as long as 10 weeks in those left untreated, vasodilatory treatment is commonly used [4].

## 2. Case Presentations

**Case 1**

**Figure 1:**
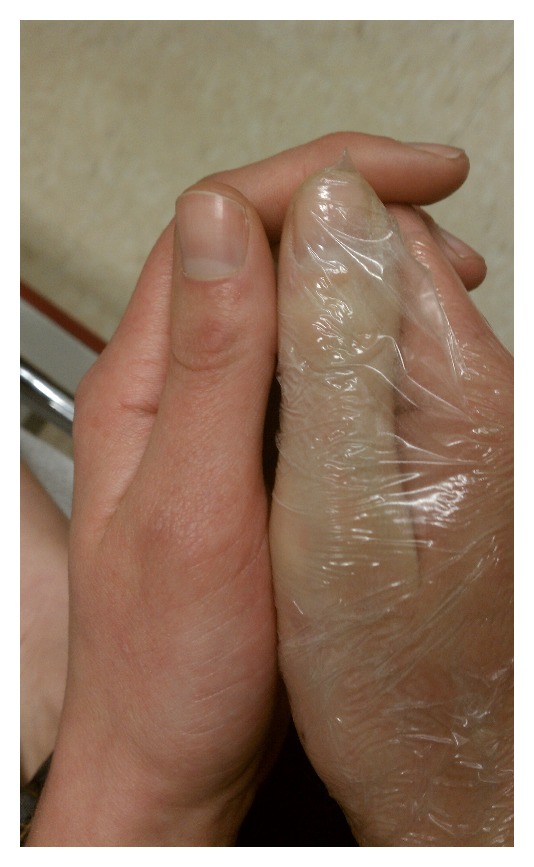
Case 1—right thumb 20 minutes after topical application of nitroglycerin ointment and warm water bath emersion.

**Figure 2:**
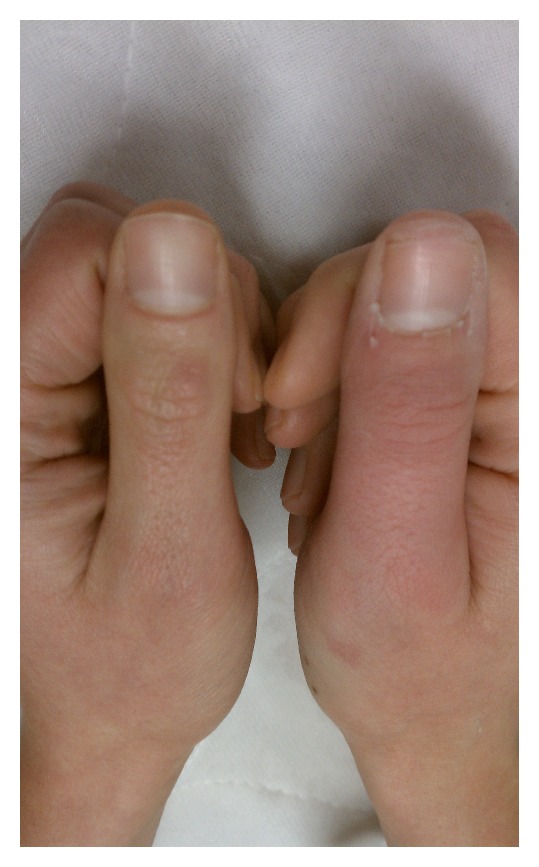
Case 1—right thumb 5 minutes after subcutaneous injections of a total of 1.5 mg of phentolamine.

A 29-year-old female presented to the emergency department approximately 30 minutes after an accidental injection of epinephrine (0.3 mg/0.3 mL, 1 : 1,000 concentration) into the pad of her right thumb. She complained of pain and numbness and noted pale coloration tracking up the palmar and dorsal surface of her right thumb. Initial therapy in the emergency department consisted of nitroglycerin ointment 2%, applied with a transparent dressing, after warm water bath soaks for approximately 20 minutes. These treatments did not change the coloration or stop the progression of symptoms (Figure 1). Phentolamine was prepared using the 10 mg/mL solution and mixing 1.5 mg (0.15 mL) with NS to a concentration of 1 mg/1 mL. Small subcutaneous injections of phentolamine were administered to each of the three locations of the thumb that included the pad and midshaft bilaterally, totaling 0.5 mg for this series of injections. This administration process was repeated for 20 and 40 minutes to a total of 1.5 mg of phentolamine. Following each series of phentolamine subcutaneous injections, thumb recoloration and some resolution of symptoms occurred within five minutes. Complete resolution of color and symptoms was noted following the third series of phentolamine injections (Figure 2). The patient was observed on telemetry for one hour following the last dose of phentolamine and was discharged home.

**Case 2**

A 41-year-old male presented to the emergency department 30 minutes after accidental injection of epinephrine (0.3 mg/0.3 mL, 1 : 1000 concentration) into the pad of his right thumb. He had numbness and blanching of his entire thumb, extending to below the metacarpal phalangeal joint, and pain with movement. Preparation of phentolamine was the same as in Case 1. Phentolamine was injected subcutaneously into the site of epinephrine injection (0.5 mg) and bilaterally at the base of the thumb (0.25 mg/injection), for a total dose of 1 mg. There was immediate improvement in color, but the digit remained cool to touch. Reassessment at 15 minutes indicated further improvement in symptoms with return of color and warmth, although numbness persisted. No further phentolamine was administered. Sensation returned 3.5 hours after phentolamine administration and the patient was discharged home.

## 3. Case Discussion

Accidental digital injection of epinephrine is uncommon. The notion that this event can cause ischemic necrosis is largely theoretical based on the mechanism of action. A review of the literature found that no cases of subcutaneous injection of epinephrine caused ischemic necrosis in those who did not receive treatment aimed at vasodilation [3–5]. Specifically, a systematic review of 59 cases of accidental injection of concentrated epinephrine (0.3 mg/0.3 mL, 1 : 1,000 concentration) into a digit reported that those without treatment (*n* = 32) had resolution of symptoms without long-term negative sequelae [4]. However pain, paresthesias, and discoloration were reported to persist in the digit for days to weeks. Due to these persistent symptoms, treatment with vasodilatory agents is commonly utilized in the emergency department. Several modalities of treatment are described and include external warming, nitroglycerin ointment, and phentolamine injection [5]. 

Since epinephrine causes vasoconstriction through agonism of alpha_1_ and alpha_2_ receptors, phentolamine is an obvious choice. Phentolamine is a competitive nonselective alpha_1_ and alpha_2_ adrenergic receptor-antagonist that is commonly used subcutaneously for the treatment of extravasation following infiltration of intravenous alpha_1_ agonist agents [6]. Extravasation from intravenous infiltration and subcutaneous injection into the digit with epinephrine presents similarly in regard to discoloration and painful symptoms. 

The current literature supports the safety and efficacy of phentolamine for use in accidental subcutaneous epinephrine injections into the digit [1, 3–5]. One case series reports 14 patients with subcutaneous epinephrine injection into a digit that received subcutaneous phentolamine at a total dose ranging from 0.5 mg to 2 mg. All patients experienced resolution of symptoms within 5 to 30 minutes [4]. Similarly, in a systematic review, 15 patients received local subcutaneous phentolamine following epinephrine injection and had complete resolution of symptoms [5]. There were no reported adverse effects from phentolamine treatment in these cases.

## 4. Conclusion

Our cases support the use of phentolamine for the treatment of subcutaneous injection of epinephrine for the rapid resolution of pain, paresthesias, and discoloration and prevention of prolonged symptoms.
